# Society Meetings

**Published:** 1915-03

**Authors:** 


### SOCIETY MEETINGS

Brief reports and announcements of meetings of societies of Western New York, and those of general scope, are requested from Secretaries. Copy should be on hand the fifteenth of the month. Full transactions will be published at cost of composition.

**DOCTOR: Is your Society properly represented here? If not, please bring our standing announcement to the attention of the members.**

### Medical Society of the State of New York.

As announced, this meeting will be held in Buffalo, April 27 to 29. Owing to the European war and the time-table of various international and national congresses, it will undoubtedly be the largest medical meeting of the year, with the exception of the A. M. A. meeting in San Francisco—and some enthusiasts do not grant even this exception.

BUFFALO is a city of between 450,000 and 500,000 inhabitants. It has about 42 square miles in area, the boundaries not having been changed for about 40 years. Owing to the inclusion within the city limits of about 1½ square miles of parks, ½ square mile of cemeteries, nearly one-third square mile of State Hospital grounds, and very large stock yards and railroad terminals and sidings, canals and docking, and the fact that ample space is provided about most dwellings, it contains comparatively little vacant territory.

The strategic importance of the site was first recognized by La Hontan in 1687. Owing to political conditions existing among the aborigines, the site of Buffalo was entirely without permanent population during the colonial period and as far back of this time as tradition goes, although there were some 25 entirely prehistoric Indian villages within the present area of Buffalo. Following Sullivan's campaign against the Iroquois in 1779, a large number of Seneca and some other Indians took refuge at Ft. Niagara and for economic reasons, these were settled about the fork of Buffalo Creek in what is now the eastern part of the city, during the following winter and spring.

The name Buffalo is said to be a corruption of the Seneca word "splitting the fork," alluding to this settlement, and has no reference to the American bison, remains of which have never been found in this region. The American town dates from about 1800; it was quite a good-sized village when it was almost entirely burned by the British in 1813; the city was incorporated in 1832.

Buffalo has more smooth pavement than any other city of the world, is one of the largest fresh-water ports in the world, its lake commerce exceeding that of all but a few ocean ports. Being at the western end of the Erie canal, it is one of the most important grain centers of the world and was the place of origin of grain elevators and hence, of elevators for transferring all kinds of merchandise. Including Tonawanda to the north it is the largest lumber market in the world. It has been said that Buffalo contains more miles of railroad track and the largest live-stock yards of any city of the world, and to have more railroads centering in it than any other city. Owing to the combination of railroads, the last statement is no longer true but it still ranks high as a live-stock and railroad center, and the railroad yards now being established to the east of the city will be the largest in the world. At one time it boasted of the largest train-shed in the world and may still claim to have the best ventilated railroad station of any large city; while, in general, its terminal facilities for passengers are most admirably planned to discourage the evil habit of loitering. New stations are, however, promised by all the principal roads. Within the last twenty years, Buffalo has become an important manufacturing center and the steel plant at Lackawanna, coterminal with Buffalo to the south, is the largest in the world. The 65th Regiment Armory, in which the meetings of the society, commercial and scientific exhibits, etc., will be held, is the largest building of the kind in the world.

Buffalo, while possessing a salubrious climate, is a windy city and it has been said that a Buffalonian can be recognized anywhere by his habit of ducking his head and grabbing his hat when turning a corner. This attribute does not apply to its inhabitants who, indeed, are more prone to seek improvement by criticism directed toward their various institutions than to boast of their advantages. We feel, however, that visitors will recognize that municipal and public services of all kinds are of a high order of efficiency. Owing to this critical spirit, Buffalo is a favorite place for trying out new plays which, if successful here, will prove so everywhere. With this exception, in regard to the method of expression of local pride and interest, Buffalo does not differ materially in its personnel, from most American cities. It is, of course, cosmopolitan, having more Poles than any other city in the world except Warsaw, a large Italian colony, and representatives of all the nations and races of the world, though comparatively few non-Caucasians.

Of special interest to the medical profession may be mentioned the following: The University of Buffalo, with medical dental, pharmacal and law departments giving complete courses and the arts department, recently organized and just on the point of reaching out beyond the first year's course; the N. Y. State Institution for the Study of Malignant Diseases; the State (insane), U. S. Marine, General and Sisters' and numerous smaller hospitals; excellent medical libraries in the Medical Dept. of the University and the (municipal) Grosvenor Library; the most liberal water supply of any city in the world, derived from Lake Erie and treated by the chlorine process; the local Health Department, co-operating with the educational and poor departments and, through its laboratories and hospitals, with the profession generally; the garbage and rubbish reclamation plants; and we may add that this journal, established by Dr. Austin Flint, one of the pioneers of experimental physiology, is surpassed in age by only two extant medical periodicals, in the western continent.

WORK OF THE COMMITTEE OF ARRANGEMENTS. The various subcommittees co-operate, through their chairmen, at weekly meetings. A good many exhibit booths have been leased and we may impress upon prospective advertisers that the concentration of all activities of the meeting except the popular lectures and banquet, under one roof, so that no attendant need leave the 65th Armory except to sleep, will render the publicity of this meeting superior to any other medical assembly that has ever been held.

As noted elsewhere, no special rates are granted by the railroads. Ample provision will be made for hotels and lodging places. A reasonable priced restaurant will be maintained in the Armory. The usual arrangements will be made for registration, issuing of badges, etc.; there will be an efficient reception committee and, in addition, the University of Buffalo will be asked to allow the students to be on hand to show visiting members and guests various courtesies in the way of information and guidance. A tea will be given for Women Physicians. The scientific program cannot yet be presented in full but it will be of high order. During the period of the meeting, the more important moving picture houses will show educational films. The schedule of popular lectures is appended.

We take this opportunity to urge a full attendance, especially of local and near-by physicians who are apt to register and hurry away. The educational value of the sessions, the scientific and the commercial exhibits, is well worth some sacrifice of convenience. We also urge that physicians not affiliated with county and state organizations avail themselves of this opportunity to learn the advantages of such affiliation. Out of a total of about 800 physicians of all schools of practice and including many not in practice at all but engaged in other business or retired, the Medical Society of the County of Erie has over 600 members. When we consider that, for the state, about 50 per cent. of all physicians are thus affiliated and for the entire country, scarcely so many, this is a noteworthy example. It should be remembered that, in addition to the membership of the County Society of unspecified practice, many others are enrolled in corresponding Homoeopathic and Eclectic organizations. But the Medical Society of the County of Erie has welcomed many who consider themselves physicians first and practitioners of a particular method, afterward. As an indication of the broad spirit now prevailing, it may be stated that the Committee of Arrangements, in reply to a question as to what action should be taken if a Homoeopathic or Eclectic School wished to make a scientific or educational exhibit, unanimously passed a resolution to draw no lines.

**Public Lectures** in connection with the 109th Annual Meeting of the Medical Society of the State of New York, in Buffalo.

Monday evening April 26: Dr. Charles J. Hastings, Medical Officer of Health, Toronto, Ontario. Subject: ''What are we doing to improve our race?''

Tuesday afternoon, April 27: Julia C. Lathrop, Chief of Children's Bureau, U. S. Department of Labor, Washington, D. C. Subject: ''Why the Children's Bureau Studies Infant mortality.''

Tuesday evening, April 27: Dr. J. W. Schereschewsky, Surgeon, Public Health Service, Washington, D. C. Subject: ''The Relation of Heat to the Summer Mortality of Infants.''

Wednesday afternoon, April 28: Henry H. Goddard, Ph. D., Director, Department of Research, The Training school, Vineland, N. J. Subject: ''The Subnormal child: Who is He and What Must Be Done For Him?'' Illustrated.

Wednesday evening, April 28: Dr. Thomas Darlington, American Iron & Steel Institute, New York. Subject: ''Welfare Work in Industry.'' Illustrated.

Thursday afternoon, April 29: Edward M. VanCleave, Managing Director, National Committee for the Prevention of Blindness, New York. Subject: ''Saving Sight and Saving Citizens.'' Illustrated.

Thursday evening, April 29: George S. Barrows, Philadelphia, representing the Illuminating Engineering Society. Subject: ''Right and Wrong Methods of Interior Illumination.'' Illustrated.

Last title subject to change. F. W. BARROWS.

---

The annual meeting of the **Gross Medical Club** was entertained by Dr. James A. MacLeod at the Hotel Touraine, Friday February 19; Dr. A. G. Bennett, president in the chair. A very interesting paper, "Air and Rhinitis" was presented by Dr. Walter M. Wurtz of Tonawanda; it was lengthily discussed by Drs. Forsythe and Cott. On the resolution of Dr. W. H. Baker of Williamsville, that the present law regarding the per-capita tax payment of the fees of Medical officers of towns and villages of N. Y. State be left as it is. A very animated discussion took place which ended by a motion of Dr. Dowd, that the Gross Medical Club go on record as endorsing the resolution: it was carried. Several most interesting cases were reported by Drs. Cotts, Bennett, McKinney and Phelps of East Aurora.

The annual election resulted in the election of: President, Dr. J. Henry Dowd; vice-president, Dr. W. Warren Britt; secretary, Dr. Chester C. Cott. A banquet for the members and guests followed the business meeting.

---

The regular meeting of the **Medical Society of the County of Erie** was held at the Municipal Hospital, 770 East Ferry Street, Buffalo, Monday evening February 15, 1915. The attendance was unusually large. Before the meeting an opportunity was given to inspect the several branches of the hospital.

President Hurd called the meeting to order at 9 o'clock. The minutes of the annual meeting held December 21, 1914, were approved as published. The minutes of the council meeting held January 18, 1915 were read by the secretary and approved. Dr. Jacobs, chairman of the committee on membership, presented the names of the following applicants who were duly elected: Dr. R. C. Conklin, Dr. Hugh B. Deegan, and Dr. Carl G. Leo-Wolf.

A resolution, offered by Dr. Julius Richter, was adopted directing the secretary to communicate with the Academies of Medicine in Western N. Y., requesting their co-operation for the 109th annual meeting of the New York State Medical Society to be held in Buffalo in April.

The following program was then carried out with ten minute papers and five minute discussions:

"Defects in the Present Hospital Methods of Treating Pulmonary Tuberculosis." Dr. Henry C. Buswell. Discussion by Dr. DeLancey Rochester and Dr. Harry Mead.

"Report of Surgical Tuberculosis in Children Treated at the Municipal Hospital, Department of Health, Buffalo, N. Y." Dr. Ward W. Plummer. Discussion by Dr. Carl G. Leo-Wolf.

"Why Municipalities Should Provide Facilities for Treating Urolcgic Patients." Dr. Thos. B. Carpenter. Discussion by Dr. James Gardner and Dr. Nelson W. Wilson.

"Crossed and Mixed Infections in Acute Communicable Diseases." Dr. Nelson G. Russell. Discussion by Dr. E. L. Beebe.

"Prophylaxis and Treatment of Otitis media in Acute Communicable Diseases." Dr. John Fairbairn. Discussion by Dr. Clayton M. Brown.

"A Plea for Greater Laboratory Facilities in the Department of Health, Buffalo, N. Y. Dr. William G. Bissell.

After concluding the program a collation was served.

The following preamble and resolution, offered by Dr. A. H. Briggs, was adopted:

WHEREAS the laboratory of the Health Department has been and is of untold value to the physicians of the city in assisting in the diagnosis and prevention of disease, and

WHEREAS the laboratory facilities of the Department are shown to be taxed to their utmost, as outlined in a report recently submitted by the Bacteriologist to the Health Commissioner, and

WHEREAS Buffalo should keep pace with other large cities of its size in regard to furnishing its physicians laboratory facilities, particularly to be used for the benefit of the poor, be it.

RESOLVED, that the Erie County Medical Society sanction and approve of the action of the Health Commissioner in his present efforts to obtain more help, larger quarters and sufficient appropriations for this class of health department administration.

---

**The Central and Northern New York Alumni Association of The University of Buffalo** held its first annual meeting and banquet in Syracuse, February 10. 65 graduates representing all departments attended. Dr. Archer D. Babcock of Syracuse was toastmaster. Dr. George F. Cott, Dr. Abraham Hoffman, Dean Willis G. Gregory, M. D., and Dr. Lesser Kauffman, all of Buffalo, were among the speakers.

---

**The Federated Alumni Association of The University of Buffalo,** held its organization meeting and dinner at the Hotel Statler, Buffalo, February 22. A more extended notice will appear in our next issue.

---

**The Buffalo Academy of Medicine** has held the following meetings since our last notice: January 27, Section of Pathology, Present Status of our Knowledge of the Aetiology and Distribution of Typhus Fever; Dr. John F. Anderson, Director of Hygienic Laboratory, U. S. P. H. S., Washington. Discussion opened by Major W. W. Quinton, M. D., Buffalo.

February 3, Section of Surgery, Limitations in Modern Diagnosis in Urology; Dr. Victor C. Peterson, New York.

February 10, Medical Section, Tobacco Heart; Dr. Harlow Brooks of New York.

February 17, Section of Obstetrics and Gynaecology, Experiences as Obstetric Consultant, Dr. Peter W. Van Peyma; The Physician and Midwife as Factors in Puerperal Infections, Dr. Nahum Kavinoky; Presentation of Large Ovarian Cyst, Dr. Charles R. Borzilleri, all of Buffalo.

---

**The Elmira Academy of Medicine** held its regular monthly meeting January 30. Dr. Charles G. R. Jennings gave a paper on the Anatomy and Physiology of Pituitary Gland and Dr. LaRue Colegrove on Some Clinical Manifestations of the Derangement of the Pituitary Gland.

---

**The Rochester Academy of Medicine,** Section II, held its regular meeting February 10. The following papers were presented: Treatment of Hyperthyroidism, Dr. N. W. Soble; Parathyroids and Tetany, Dr. E. L. Hanes; Therapeutic Use of Parathyroid Substance, Dr. W. T. Mulligan.

---

**The Rochester Pathological Society** at its regular meeting of January 28, listened to a paper by Dr. E. W. Ruggles on Extra genital and Innocent Syphilis; and on February 11, to one by Dr. J. R. Culkin on the Differential Diagnosis of Fevers.

---

**The Alumni Association of the Dental Department of The University of Buffalo** held its 15th annual clinic in Buffalo, February 5 and 6. The dinner was on the evening of the 5th. The faculty invited the members and guests to inspect the college building and the recently installed radiograph and served luncheon on February 6th.

